# The combined signatures of G protein-coupled receptor family and immune landscape provide a prognostic and therapeutic biomarker in endometrial carcinoma

**DOI:** 10.1007/s00432-023-05270-4

**Published:** 2023-08-16

**Authors:** Shengyue Chen, Xukai Luo, Baicai Yang, Jingming Zhuang, Jinshuai Guo, Yingjie Zhu, Jiahang Mo

**Affiliations:** 1 Dalian Medical University, Dalian, Liaoning China; 2 Obstetrics and Gynecology Hospital, Fudan University, Shanghai, China; 3 Jiaxing University Affiliated Women and Children Hospital, Jiaxing, China; 4 Shanghai General Hospital, Shanghai Jiao Tong University School of Medicine, Shanghai, China

**Keywords:** Endometrial carcinoma, G protein-coupled receptor, Tumor microenvironment, Immunotherapy, Prognosis

## Abstract

G protein-coupled receptors (GPRs) are one of the largest surface receptor superfamilies, and many of them play essential roles in biological processes, including immune responses. In this study, we aim to construct a GPR- and tumor immune environment (TME-i)-associated risk signature to predict the prognosis of patients with endometrial carcinoma (EC). The GPR score was generated by applying univariate Cox regression and the Least Absolute Shrinkage and Selection Operator (LASSO) Cox regression in succession. This involved identifying the differentially expressed genes (DEGs) in the Cancer Genome Atlas-Uterine Corpus Endometrioid Carcinoma (TCGA-UCEC) cohort. Simultaneously, the CIBERSORT algorithm was applied to identify the protective immune cells for TME score construction. Subsequently, we combined the GPR and TME scores to establish a GPR-TME classifier for conducting clinical prognosis assessments. Various functional annotation algorithms were used to conduct biological process analysis distinguished by GPR-TME subgroups. Furthermore, weighted correlation network analysis (WGCNA) was applied to depict the tumor somatic mutations landscapes. Finally, we compared the immune-related molecules between GPR-TME subgroups and resorted to the Tumor Immune Dysfunction and Exclusion (TIDE) for immunotherapy response prediction. The mRNA and protein expression of GPR-related gene P2RY14 were, respectively, validated by RT-PCR in clinical samples and HPA database. To conclude, our GPR-TME classifier may aid in predicting the EC patients’ prognosis and immunotherapy responses.

**Supplementary Information:**

The online version contains supplementary material available at 10.1007/s00432-023-05270-4.

## Introduction

Endometrial carcinoma (EC) is the sixth most prevalent cancer in women, with over 400,000 new diagnoses made in 2020, according to global cancer statistics (Sung et al. 2021). Though many early-stage EC cases are cured with surgery alone, there are a notable number of women with aggressive variants whose prognosis remains dismal (Crosbie et al. 2022; Oaknin et al. 2022a). Traditionally, EC was subclassified into two types based on its clinical and pathological features. Type I ECs are more common and result from unopposed estrogenic stimulation of the endometrium (Bokhman 1983). The histotypes of type I ECs are mostly endometrioid with indolent biological behavior, accompanied by PTEN and KRAS mutations and microsatellite instability (MSI) status aberrance (Huvila et al. 2021). In contrast, type II ECs are aggressive phenotypes and are high-grade carcinomas. This subtype is primarily identified by p53 mutations and is considered the cause of relapse and death. (Brinton et al. 2013).

In 2013, The Cancer Genome Atlas (TCGA) Research Network proposed molecular subtypes of EC based on genomic profiles. These subtypes are the POLEmut group, MMRd (hypermutated/microsatellite unstable) group, SMP (no specific molecular profile) group, and p53abn (serous-like) group (Kandoth et al. 2013). Other than significant molecular abnormalities, these molecular subtypes differ in terms of genetic and environmental risk factors, prognosis, and response to hormonal therapy (Huvila et al. 2021). Surrogate markers for these molecular subtypes have been developed and successfully implemented in clinical practice (Talhouk et al. 2015, 2017; Stelloo et al. 2016). However, challenges remain in current EC classification systems, particularly in prognostic and therapeutic evaluation (McAlpine et al. 2018). Thus, new molecular patterns defining prognosis and therapeutic response in EC are urgently needed.

The G protein-coupled receptors (GPRs) family, a large superfamily of cell-surface signaling proteins, is involved in a variety of biological processes, including cell adhesion and motion (Orduna-Castillo et al. 2022), metabolites signaling transduction (Uranbileg et al. 2022; Brown et al. 2020; Pillai et al. 2022), and even immune responses (Ge et al. 2020). Aberrant GPR expression has been observed in various cancers (Bar-Shavit et al. 2016). Recent GPRs function research has highlighted mechanisms related to metabolites among these carcinous patterns. As a metabolic response to acid stress, the acid-sensing GPR (GPR68) has been reported to mediate lipogenesis in cancer cells, thereby promoting lipid droplet accumulation and enhancing viability under acidic stress (Pillai et al. 2022). In human hepatocellular carcinoma (HCC), S1P lyase (SPL) converts sphingolipids into glycerophospholipids (LPI and LPG). These subsequently combine with GPR55 and activate the p38/MAPK pathway, contributing to tumor progression (Uranbileg et al. 2022). Other metabolite-related GPRs, including lactate receptor (GPR81) (Brown et al. 2020), succinate receptor‑1 (GPR91) (Kuo et al. 2022), neurokinin-1 receptor (Zhang et al. 2022), and purinergic receptor (Wang et al. 2020), were extensively investigated in cancer research. In ECs, estrogenic transmembrane receptor (GPR30) has been extensively studied over the decade (He et al. 2009). Numerous evidence has already revealed that estrogen-mediated GPR signaling played a critical role in gaining malignant phenotypes (He et al. 2012; Zhang et al. 2019). In light of the preceding, it is necessary to propose a novel GPR molecular set that can shed light on EC prognosis and biological behavior.

Interestingly, numerous GPRs have been studied for their immunological functions. Activation of A2A receptors (A2AR), a typical GPR with a high affinity for adenosine, has been shown to promote the immune escape of cancer cells in tumor niche (Sun et al. 2022). The lactate receptor (GPR81) in breast cancer nidus promotes tumor growth through a paracrine mechanism involving dendritic cell (DCs) function impairment. This paracrine mechanism is complementary to an autocrine mechanism by which lactate induces programmed cell death ligand 1 (PD-L1) in tumor cells via activation of GPR81; therefore, inhibition of GPR81 signaling may provide a novel cancer immunotherapy strategy (Brown et al. 2020). Activated by low pH, proton-sensing GPR has been reported to promote PD-L1 expression in tumor cells (Mori et al. 2021). GPR87 expression is positively correlated with immune infiltration in lung adenocarcinoma (LUAD), suggesting potential benefits from immune checkpoint inhibitors (ICIs) (Bai et al. 2022). Another GPR, lysophosphatidic acid receptor 6 (LPAR6), was excluded from the immune infiltration evaluation (He et al. 2022, 2021). Therefore, given that ICIs agents are widely investigated for treating ECs (Ott et al. 2017; Makker et al. 2019), a series of biomarkers linking GPRs and immune features are ready for clinical application.

## Materials and methods

### Data sources and available analysis platforms

The current study enrolled 533 EC samples with RNA sequencing data and clinical information from TCGA (https://portal.gdc.cancer.gov/). Moreover, single-cell RNA (scRNA) sequencing data with cell reannotation of 1 microsatellite instability-high/mismatch repair-deficient (MSI-H/MMR-d) EC was obtained from GSE193430 (https://www.ncbi.nlm.nih.gov/geo/query/acc.cgi?acc=GSE193430) to visualize GPR scores in each immune cell (Guo et al. 2022). External validation was performed on an EC database comprised of 95 patients from the Clinical Proteomic Tumor Analysis Consortium (CPTAC) as described by Li et al. (three were excluded for data deficient) (Dou et al. 2020; Li et al. 2023). Another Gene Expression Omnibus (GEO) dataset for differentially expressed genes (DEGs) seeking was obtained from GSE17025 (https://www.ncbi.nlm.nih.gov/geo/query/acc.cgi) (Day et al. 2011). The extended analysis utilized four of the available analysis platforms. The Metascape (https://metascape.org) was applied for gene set annotation analysis (Zhou et al. 2019); Tracking Tumor Immunophenotype (TIP; http://biocc.hrbmu.edu.cn/TIP/) for immune evolutionary analysis (Xu et al. 2018); Tumor Immune Dysfunction and Exclusion (TIDE; http://tide.dfci.harvard.edu/) for immunotherapy response prediction (Jiang et al. 2018); and Proteomaps (https://www.proteomaps.net/) for depicting the protein composition of different subgroups (Liebermeister et al. 2014). Lastly, the Human Protein Atlas (HPA) (https://www.proteinatlas.org) was searched for P2YR14 immunohistochemical (IHC) staining assessment between normal and EC tissues (Liu et al. 2021a).

### GPR-related genes and tumor immune microenvironment cells quantification

A compendium of 872 GPR-related genes was acquired from the Molecular Signatures Database (MSigDB; http://www.gsea-msigdb.org/gsea/msigdb) (Liberzon et al. 2015). Expression of all GPR-related genes was extracted from the Cancer Genome Atlas-Uterine Corpus Endometrioid Carcinoma (TCGA-UCEC) cohort. The CIBERSORT algorithm (https://cibersort.stanford.edu/) (Chen et al. 2018) was applied to quantify 22 immune cells based on the transcriptomes of the TCGA-UCEC cohort for tumor immune microenvironment (TME-i) cells.

### Establishment of GPR score, TME score, and GPR-TME classifier

DEGs of GPR between ECs and normal uterine tissue were first extracted for subsequent analysis (“limma” package, version 3.50.3; Log (FC) > 1 or <  − 1, *P* < 0.05 were regard as the cutoff). The prognostic assessment of GPR-related genes and TME-i cells was further narrowed by univariate Cox regression analysis in the TCGA-UCEC cohort (“survminer” R package; version 0.4.9; with a cutoff of *P* < 0.05). Five protective immune cells were isolated for TME score construction, and 20 GPR-related genes were identified using the Least Absolute Shrinkage and Selection Operator (LASSO) regression model to generate a GPR risk signature (GPR score). An advanced algorithm, “bootstrap,” was applied to stabilize the coefficient generated from LASSO in both the GPR and TME score constructions. Simply, the GPR score was assigned by.

$$\mathrm{GPR score}={\sum }_{i=1}^{20}Xi\times Yi$$.

Likewise, the TME score was given by.

$\mathrm{TME score}={\sum }_{j=1}^{5}Xj\times Yj$.where $Xi$ is the relative expression value of each selected gene, $Yi$ is the coefficient modified by the “bootstrap” algorithm; $Xj$ is the relative expression value of each selected immune cell, and $Yj$ is the coefficient modified the same algorithm. The GPR and TME scores were then integrated to develop the GPR-TME classifier. Subsequently, EC samples were divided into the following subgroups: GPR_high + TME_low, intermediate mixed (GPR_high + TME_low and GPR_low + TME_low), and GPR_low + TME_high based on the mean value of GPR score and TME score in TCGA-UCEC cohort.

### Single-sample gene set enrichment analysis and fast gene set enrichment analysis

The “clusterProfiler” package (version 4.4.4) was used for single-sample gene set enrichment analysis (ssGSEA) to clarify the pathways enriched in different subgroups based on the expression level of GPR or TME-i cells (Liu et al. 2020). After combining the intermediate subgroups, the “fgsea” (version 1.20.0) and the “msigdbr” (version 7.5.1) packages were applied for fast GSEA analysis.

### Comprehensive analysis of GPR score in a scRNA sequencing set

A unique sample of scRNA sequencing data (MSI‑H/MMR‑d EC) containing only immune cells was enrolled for GPR expression analysis among these infiltrative cells. The “Seurat” (version 4.3.0), “tidyverse” (version 1.3.2), and other subordinate packages were used to visualize the abundance of GPR scores in TME-i cells. Furthermore, the “CellChat” package (version 1.6.1) was used to outline intercellular communication.

### Weighted correlation network analysis based on GPR-TME classifier

Weighted correlation network analysis (WGCNA) can be used to identify clusters of highly correlated genes and summarize them using the module eigengene or an intramodular hub gene (Langfelder and Horvath 2008). Thus, the “WGCNA” R package (version 1.71) was used to generate distinct modules (sft value was set as 0.90; Supplementary Fig. 1A). Then the desired module (marked “yellow”) was identified, and the gene clusters belonging to this module were submitted to Metascape for functional analysis.

### Tumor somatic mutation, functional annotation, and TIP analysis

Somatic mutation data was available in the TCGA- UCEC database. The top 20 mutation genes were obtained using the “maftools” package (4.1.2) and then compared between GPR-TME subgroups. Hub genes with significant differences among GPR-TME subgroups were then extracted and analyzed. As described previously (Liu et al. 2022), each EC case's tumor mutation burden (TMB) score was also calculated. DEGs analysis was repeated before submitting to Proteomaps for functional annotation. Ten cases of each GPR-TME subgroup (including the mix subgroup) were randomly sampled to generate a matrix before TIP analysis. The returned online tool data were then visualized using the “pheatmap” R package (version 1.0.12).

### Quantitative real time PCR

Tissue RNA Purification Kit PLUS (EZBiosciences, China) was utilized to extract RNA from EC tissue samples (twelve pairs). After extraction, NanoDrop 2000 spectrophotometer (Thermo Scientific, USA) was used to detect RNA quantity and concentration. Hifair^®^ III Reverse Transcriptase Kit (YEASEN, China) was subsequently designed for reverse transcription of total RNA to cDNA. Quantitative real time PCR (qRT-PCR) was conducted using the Taq Pro Universal SYBR qPCR Master Mix Kit (Vazyme, China). Record the cycling threshold (Ct) for P2RY14 and calculate the P2RY14 mRNA expression in cancer and paracancerous tissues with the 2^−ΔΔCt^ method. All steps of the qRT-PCR were performed according to the reagent instructions and all experiments were repeated three times. The primers used in qRT-PCR protocol are presented in Table 1.

**Table 1:** The primers used in qRT-PCR

Gene	Forward (5′–3′)	Reverse (5′–3′)
P2RY14	AGCTGAACGTGTTTGTGTGC	GGAACAGCAAGGAGGAGCAT
GAPDH	GACTTCAACAGCAACTCCCAC	TCCACCACCCTGTTGCTGTA

### Statistical analysis

All statistical analysis was performed in R (version 4.1.0). Standard tests included the Student’s *t* test, Wilcoxon rank-sum test, and Kruskal–Wallis test. Spearman correlation analysis (“ggcorrplot” R package; version 0.1.4) was used to determine the relationship between the GPR-related genes/TME-i cells. The log-rank test and Cox regression were used to investigate related independent patients’ prognosis classifiers. *P* < 0.05 was considered statistically significant.

## Results

### Development of the GPR score based on GPR-related genes in TCGA-UCEC and validation in CPTAC

**Fig. 1:**
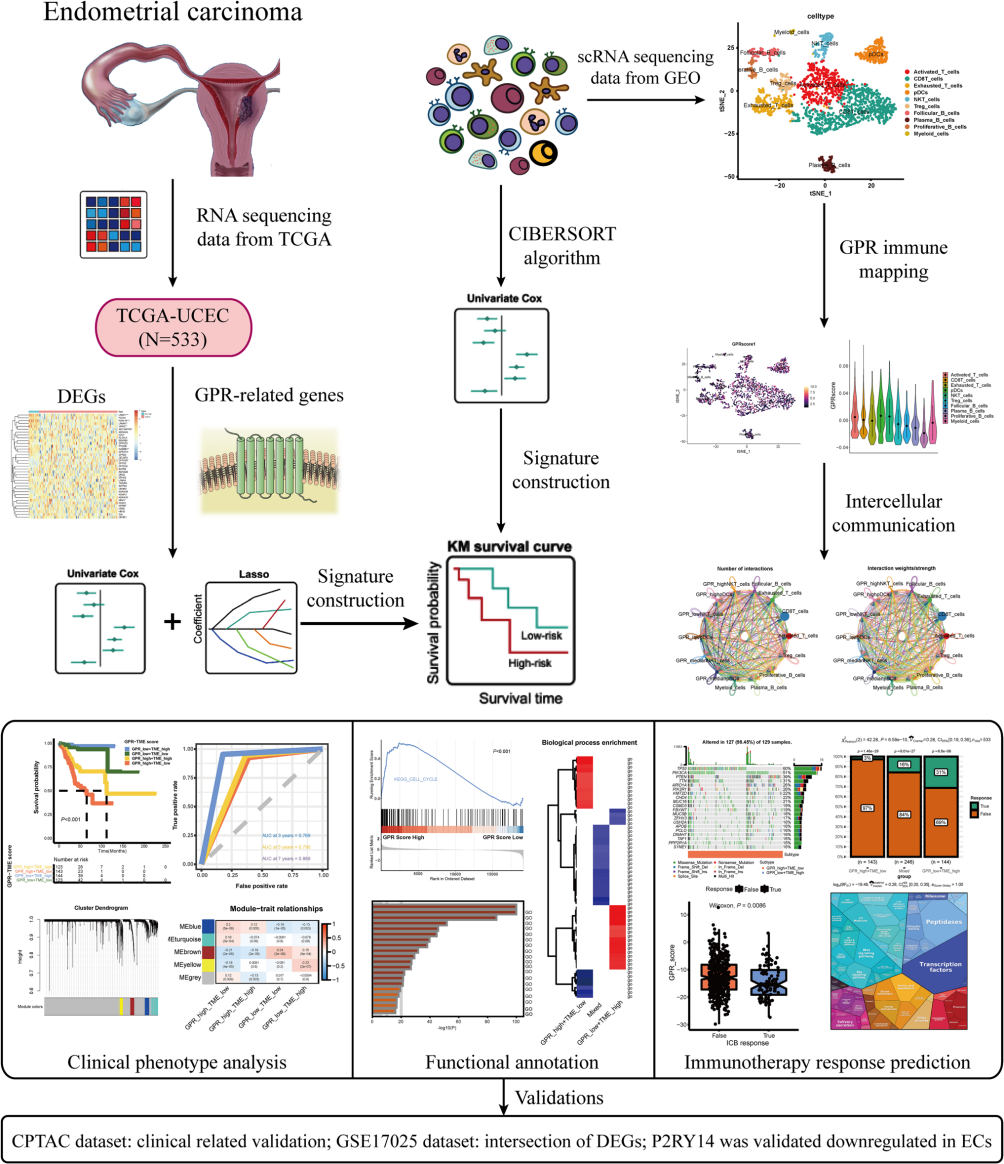
Schematic diagram portraying the establishment and comprehensive analysis of the GPR-TME classifier. *TCGA-UCEC* the Cancer Genome Atlas-Uterine Corpus Endometrioid Carcinoma, *DEGs* differentially expressed genes, *GPR* G protein-coupled receptor, *GEO* Gene Expression Omnibus

**Fig. 2:**
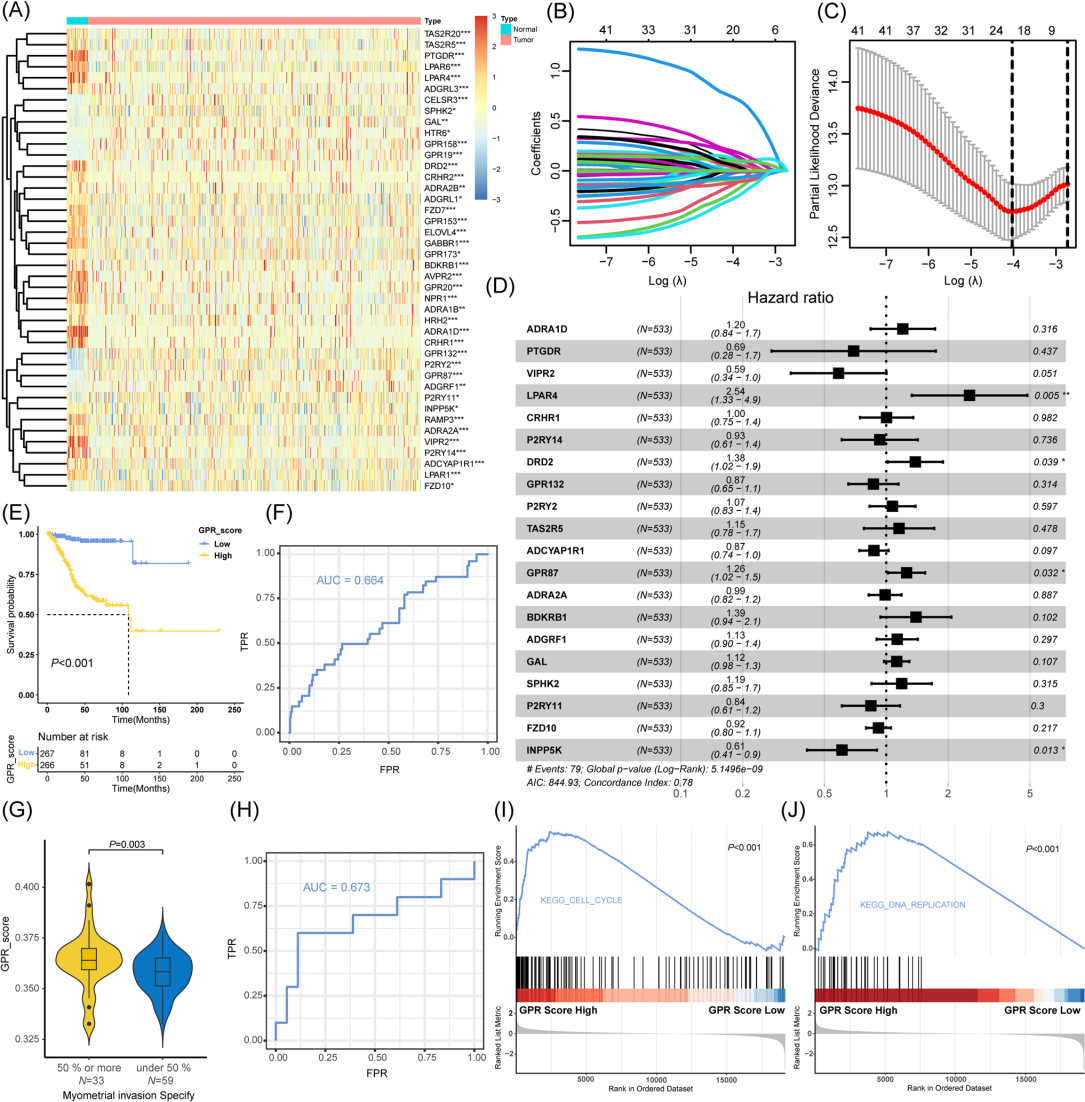
Development of the GPR score based on GPR-related genes in TCGA-UCEC. **A** Heatmap shows DEGs and prognostic genes candidates for GPR score construction. **B** Selection of the optimal λ in the LASSO analysis. **C** LASSO coefficient profiles of 20 genes in TCGA-UCEC cohort. **D** Forest plot shows a multivariate Cox analysis of these enrolled genes. **E** K–M curves for the OS of EC patients in the low- and high-risk subgroups based on the GPR score. **F** ROC curves demonstrate the predictive efficiency of the GPR score in the TCGA cohort. **G** Validation of the predictive role of GPR score in CPTAC cohort. **H** ROC curves demonstrate the predictive efficiency of the GPR score in the CPTAC cohort. **I, J** GSEA identifies the phenotype differences between the GPR score high and GPR score low subgroups. Significance: *P < 0.05; **P < 0.01. *GPR* G protein-coupled receptor, *TCGA-UCEC* the Cancer Genome Atlas-Uterine Corpus Endometrioid Carcinoma, *DEGs* differentially expressed genes, *LASSO* Least Absolute Shrinkage and Selection Operator, *K–M* Kaplan–Meier, *OS* overall survival, *EC* endometrial carcinoma, *ROC* receiver operating characteristic curves, *CPTAC* Clinical Proteomic Tumor Analysis Consortium, *GSEA* gene set enrichment analysis

The schematic diagram of the entire study is depicted in Fig. 1. To develop a method indicative of GPR expression, 533 EC cases from TCGA-UCEC were enrolled. Figure 2A depicts a preliminary screen of DEGs and prognostic genes (univariate Cox analysis, FDR < 0.05). Then, the LASSO regression analysis was performed to narrow down the most robust prognostic genes among the candidate genes by evaluating their risk prediction contributions (Fig. 2B, C). Additional results of principal component analysis (PCA) also demonstrate the different clusters based on the selected risk genes (Supplementary Fig. 1D, E). As shown in Fig. 2D, a multivariate Cox analysis of these genes revealed that 20 GPR-related genes were ultimately involved in GPR score construction. Based on the mean value of the GPR score in the TCGA-UCEC cohort, two subgroups of EC patients were identified. Statistically, patients with low GPR scores had a longer survival rate than patients with high GPR scores (Fig. 2E). The receiver operating characteristic (ROC) curves demonstrated the value of survival prediction with a total area under the curve (AUC) of 0.664 (Fig. 2F). Considering the limited prognostic data regarding ECs, the recognized phenotype “myometrial invasion” was applied for prognostic assessment. In an external cohort, the GPR score was validated as a risk factor for myometrial invasion in ECs (Fig. 2G); and its predictive role for myometrial invasion in the CPTAC cohort was comparable to the former (Fig. 2H; AUC = 0.673 vs. AUC = 0.664). EC samples with higher GPR scores were also significantly enriched for the proliferative phenotype (Fig. 2I, J). In this section, a GPR score for predicting the prognosis of TCGA-UCEC patients was developed, and the underlying function of GPR molecules in ECs was examined.

### Development of the TME score and correlation between GPR score and TME cells

**Fig. 3:**
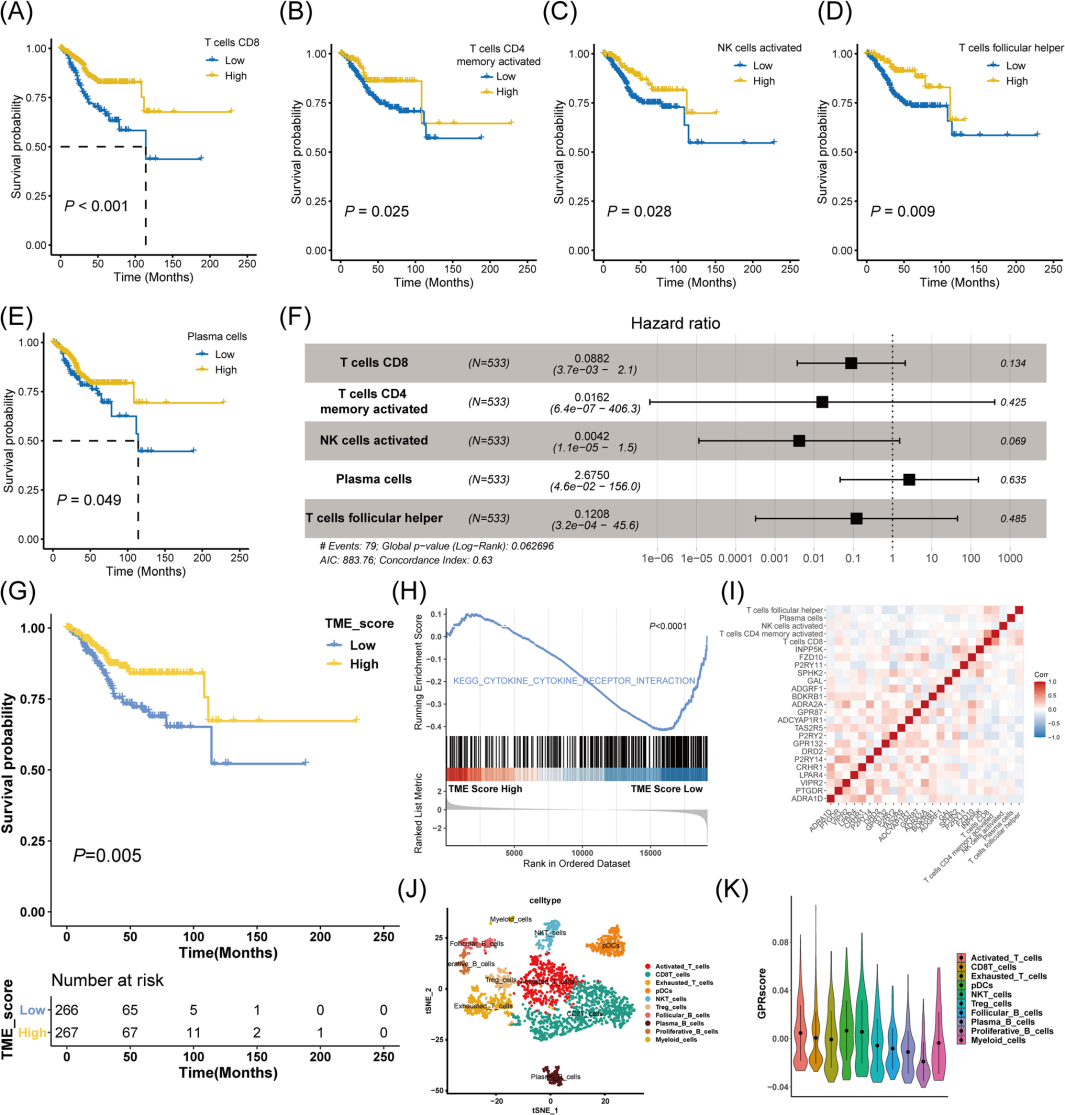
Development of the TME score and correlation analysis for GPR score and TME cells. K–M curves for the OS of EC patients in low- and high-CD8 T cell **A** memory activated CD4 T cell **B** activated NK cell **C** follicular helper T cell **D**, and plasma cell **E** subgroups. **F** Forest plot shows a multivariate Cox analysis of these immune cells. **G** K-M curves for the OS of EC patients in the low- and high-TME score subgroups. **H** GSEA identifies the phenotype differences between the TME score high and TME score low subgroups. **I** Correlation analysis shows the relationship between the GPR and TME score components. **J** Immune cell clusters of MSI-H/MMR-d EC. **K** GPR score counted in each immune cell cluster. *TME* tumor environment, *GPR* G protein-coupled receptor, *K–M* Kaplan–Meier, *OS* overall survival, *EC* endometrial carcinoma, *CD* cluster of differentiation, *NK* natural killer, *GSEA* gene set enrichment analysis, *MSI-H/MMR-d* microsatellite instability-high/mismatch repair-deficiency, *pDCs* plasmacytoid DCs, *NKT* natural killer T, *Treg* regulatory T cell

The CIBERSORT algorithm was applied to quantify 22 immune cells based on transcriptomes of the TCGA-UCEC cohort to generate a TME-based signature. Subsequently, five types of immune cells were identified for their protective roles in overall survival (OS) based on their respective optimum cut-off value. These immune cell types include CD8 T cells, memory-activated CD4 T cells, activated natural killer (NK) cells, follicular helper T (Tfh) cells, and plasma cells (Fig. 3A–E). Figure 3F depicts the multivariate Cox analysis of these immune cells. Contrary to the GPR score, patients with high TME scores showed statistically longer survival than patients with low TME scores (Fig. 3G). GSEA further identified the potential differences between the TME subgroups, implying intercellular communication is more vibrant among the TME high tumors (Fig. 3H). The correlation analysis illustrates the connections between GPR expression and immune infiltrations (Fig. 3I). To further delineate the GPR score status in single cell clusters, scRNA sequencing data of MSI-H/MMR-d EC was enrolled (extra data are available in Supplementary Fig. 2). Figure 3J depicts the immune landscape of MSI‑H/MMR‑d EC. In contrast, Fig. 3K depicts the GPR score of each immune cell cluster, with plasmacytoid DCs (pDCs) and NKT presenting a seemingly higher level of GPR expression. This section established a TME score and clarified the relationship between the GPR score and immune cells.

### Prognostic value and functional annotation of the established GPR-TME classifier

**Fig. 4:**
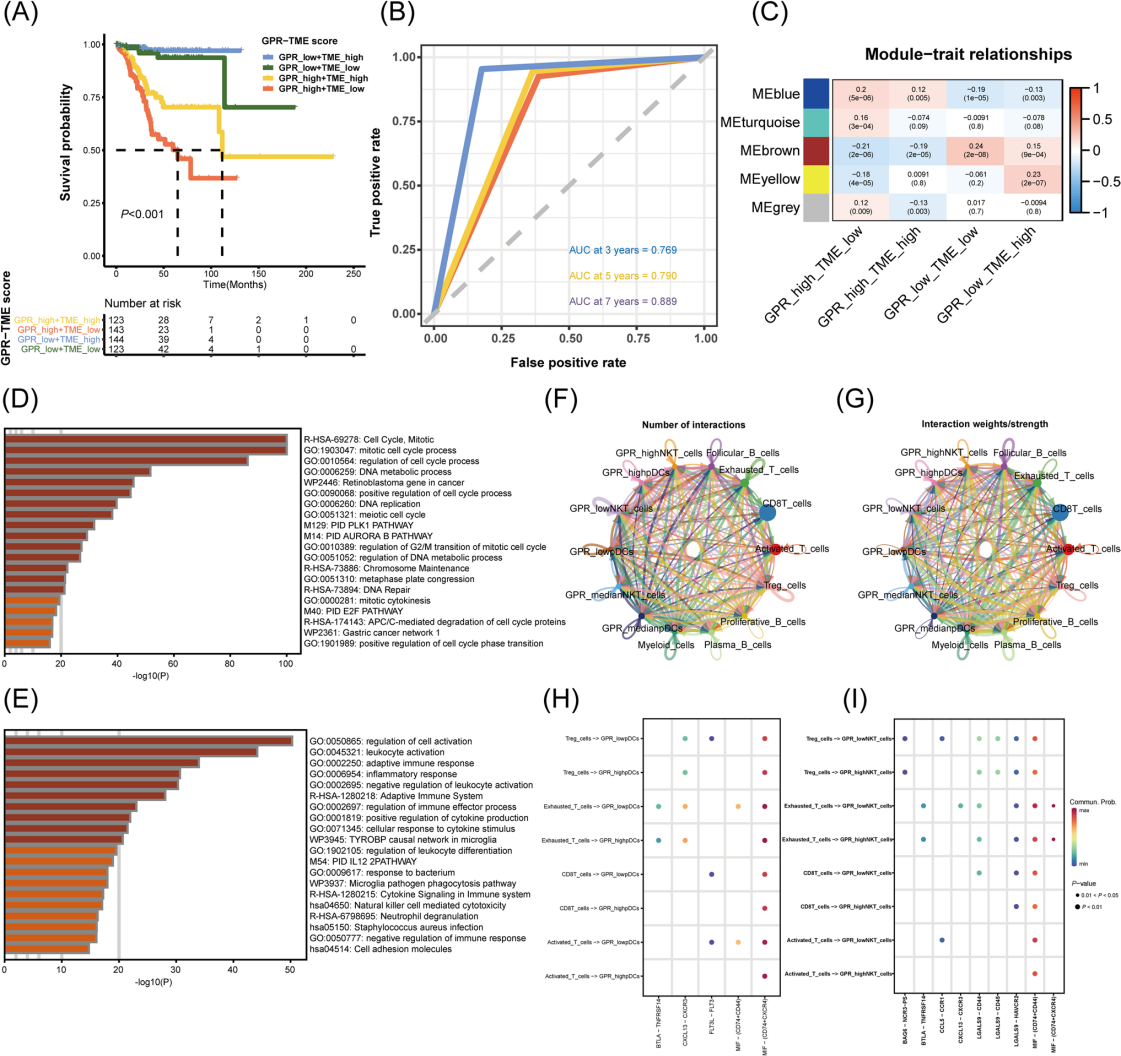
Prognostic value, cellular signaling pathways, and intercellular communication analysis based on GPR-TME classifier. **A** K–M curve for the OS of EC patients in the GRP_low + TME_high, GPR_low + TME_low, GPR_high + TME_high, and GPR_high + TME_low subgroups. **B** Time-dependent ROC curves demonstrate the predictive efficiency of the GPR-TME classifier. **C** Gene modules derived from WGCNA show the different clusters among four subgroups. **D** Top 20 annotations collected for GPR_high + TME_low subgroup. **E** Top 20 annotations collected for GRP_low + TME_high subgroup. **F** The number of interactions for intercellular communication analysis. **G** The interaction strength of intercellular communication analysis. **H** pDCs with high GPR score compromise activation of the cell chatting pathway. **I** NKT cells with high GPR score compromise activation of the cell chatting pathway. *GPR* G protein-coupled receptor, *TME* tumor environment, *K-M* Kaplan–Meier, *OS* overall survival, *EC* endometrial carcinoma, *ROC* receiver operating characteristic, *WGCNA* weighted correlation network analysis, *pDCs* plasmacytoid DCs, *NKT* natural killer T, *AUC* area under the curve

Based on the findings, we questioned whether it would be possible to combine the GPR and TME scores to construct a GPR-TME classifier for joint assessment. After combination, four subgroups were generated: GRP_low + TME_high, GPR_low + TME_low, GPR_high + TME_high, and GPR_high + TME_low. The GPR-TME classifier presented a statistically different prognosis in the TCGA-UCEC cohort (Fig. 4A). Notably, EC cases in the GRP_low + TME_high subgroup had the best prognosis compared to cases from the other three subgroups. The predictive ability of the GPR-TME classifier on OS was evaluated using time-dependent ROC curves. The AUC was 0.769 for three years, 0.790 for five years, and 0.889 for seven years, as shown in Fig. 4B. To further elucidate the significant differences in survival, WGCNA was applied for exploring the gene variations among these subgroups (Supplementary Fig. 1). The “blue” and “yellow” modules were then distinguished for the most significant variations between GPR_high + TME_low and GRP_low + TME_high subgroups (Fig. 4C). The gene clusters representing “blue” and “yellow” modules were then submitted to the online tool Metascape for functional annotation. Cell pre-filtration-related items were enriched in the GPR_high + TME_low subgroup, while immune cell activation-related items were gathered in GRP_low + TME_high subgroups (Fig. 4D, E). To determine whether GPR status could affect the function of immune cells, we analyzed intercellular communication. Based on the findings derived from Fig. 3K, pDCs, and NKT cells were selected for analysis. The number of interactions and interaction strength of intercellular communication are depicted in Fig. 4F, G, respectively. Figure 4H, I demonstrate that immune cells (pDCs and NKT cells) with high expression of GPR have less intercellular signaling activation. Overall, for this section, a GPR-TME classifier was constructed by combining GPR and TME scores. Then by using multiple bioinformatics tools, the functional differences between four subgroups were investigated.

### Simplification of the GPR-TME classifier and association between GPR-TME classifier with clinical features

**Fig. 5:**
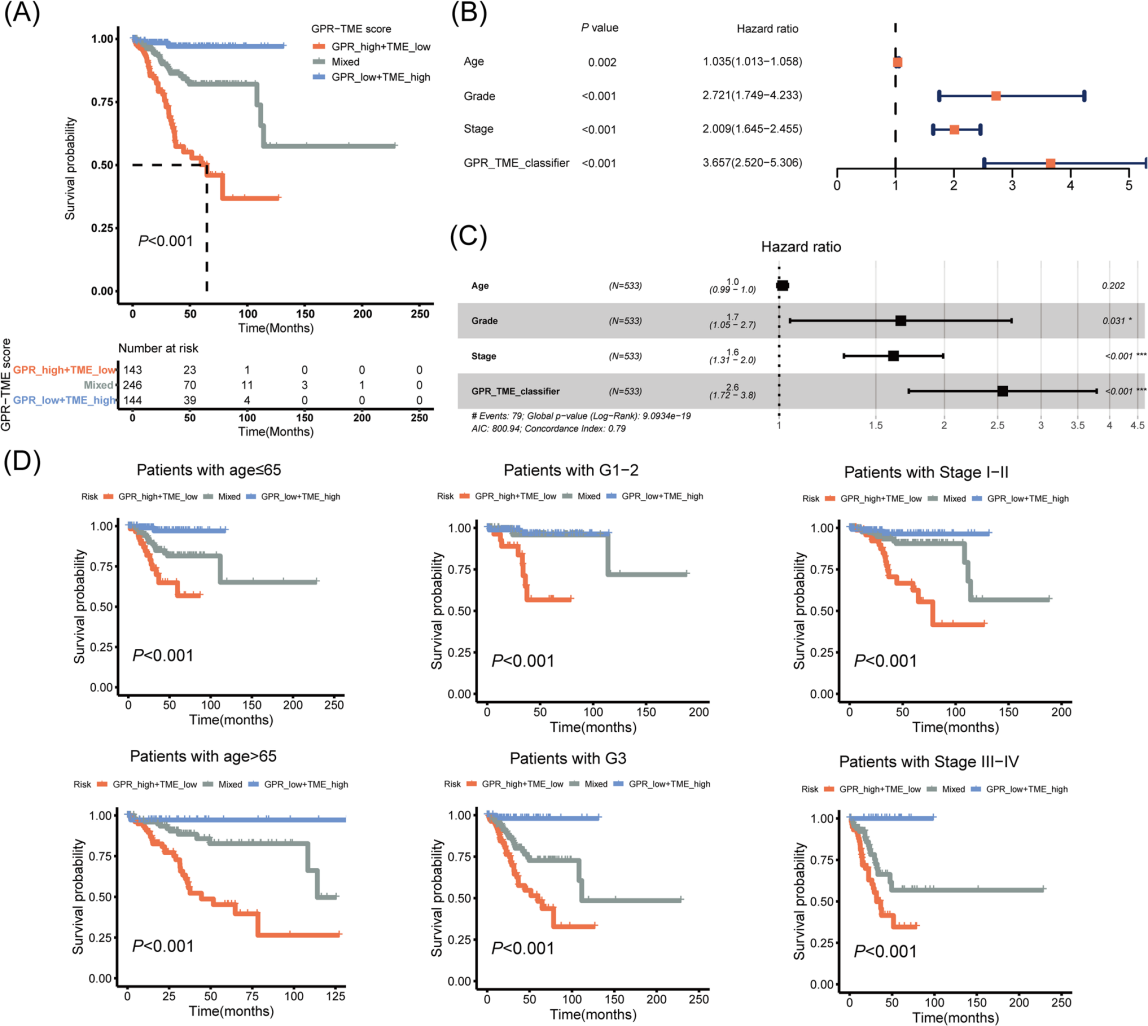
Simplification of the GPR-TME classifier for clinical application. **A** K–M curve for the OS of EC patients in the GRP_low + TME_high, Mixed, and GPR_high + TME_low subgroups. **B** Forest plot of univariate analysis shows the simplified GPR-TME classifier possesses a better-predicting efficacy than clinical parameters. **C** Forest plot of multivariate analysis shows the simplified GPR-TME classifier is an independent prognostic factor for EC patients. **D** K-M curves for the simplified GPR-TME classifier present statistically significant discriminations regardless of age, tumor grade, and stage. Significance: *P < 0.05; ***P < 0.001. *GPR* G protein-coupled receptor, *TME* tumor environment, *K–M* Kaplan–Meier, *OS* overall survival, *EC* endometrial carcinoma

**Fig. 6:**
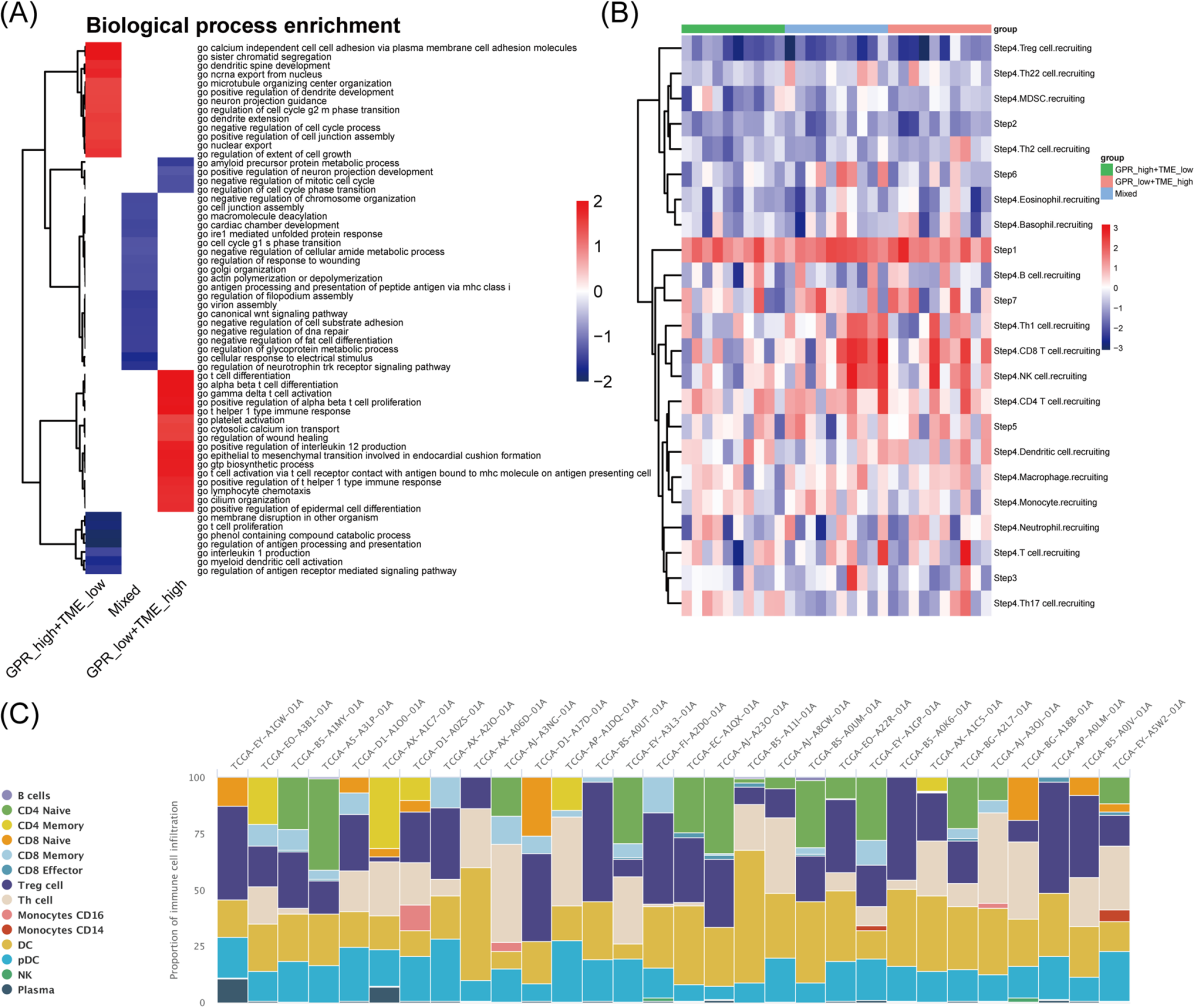
Immune characteristic of GPR-TME classifier demonstrated in different subgroups. **A** Fast GSEA for the simplified GPR-TME classifier. **B** TIP analysis for the simplified GPR-TME classifier. **C** Fourteen immune cells infiltration atlas of individual patient (*n* = 30) from TIP analysis. *GPR* G protein-coupled receptor, *TME* tumor environment, *GSEA* gene set enrichment analysis, *TIP* Tracking Tumor Immunophenotype

To simplify the GPR-TME classifier for clinical application, GPR_low + TME_low and GPR_high + TME_high subgroups were merged due to their less divergence. Figure 5A depicts the K-M curves for the OS of EC patients after combination. As shown in Fig. 5B, C, the GPR-TME classifier exhibited a significant correlation with the OS of EC patients (HR 2.60, 95% confidence interval (CI) 1.72–3.80; *P* < 0.001; refer to multivariate Cox analysis). These findings suggest that the GPR-TME classifier, constructed using the TCGA-UCEC cohort, was an independent prognostic factor for EC patients. Interestingly, the simplified GPR-TME classifier exhibited a quite decent predictive efficacy regardless of age, tumor grade, and stage (Fig. 5D). Fast GSEA for simplified subgroups revealed significant differences in biological process enrichment (Fig. 6A), which is similar to the Metascape functional annotation. TIP analysis was performed on an expression matrix containing ten cases from each subgroup to track the tumor immunophenotype. The TIP analysis results were then visualized using a heatmap, as shown in Fig. 6B. Fourteen immune cells infiltration atlas for individual patient (*N* = 30) are presented in Fig. 6C. We simplified the GPR-TME classifier for clinical use and clarified the relationship between the GPR-TME classifier and clinical characteristics.

### Differential patterns of tumor somatic mutations in patients among GPR-TME subgroups

**Fig. 7:**
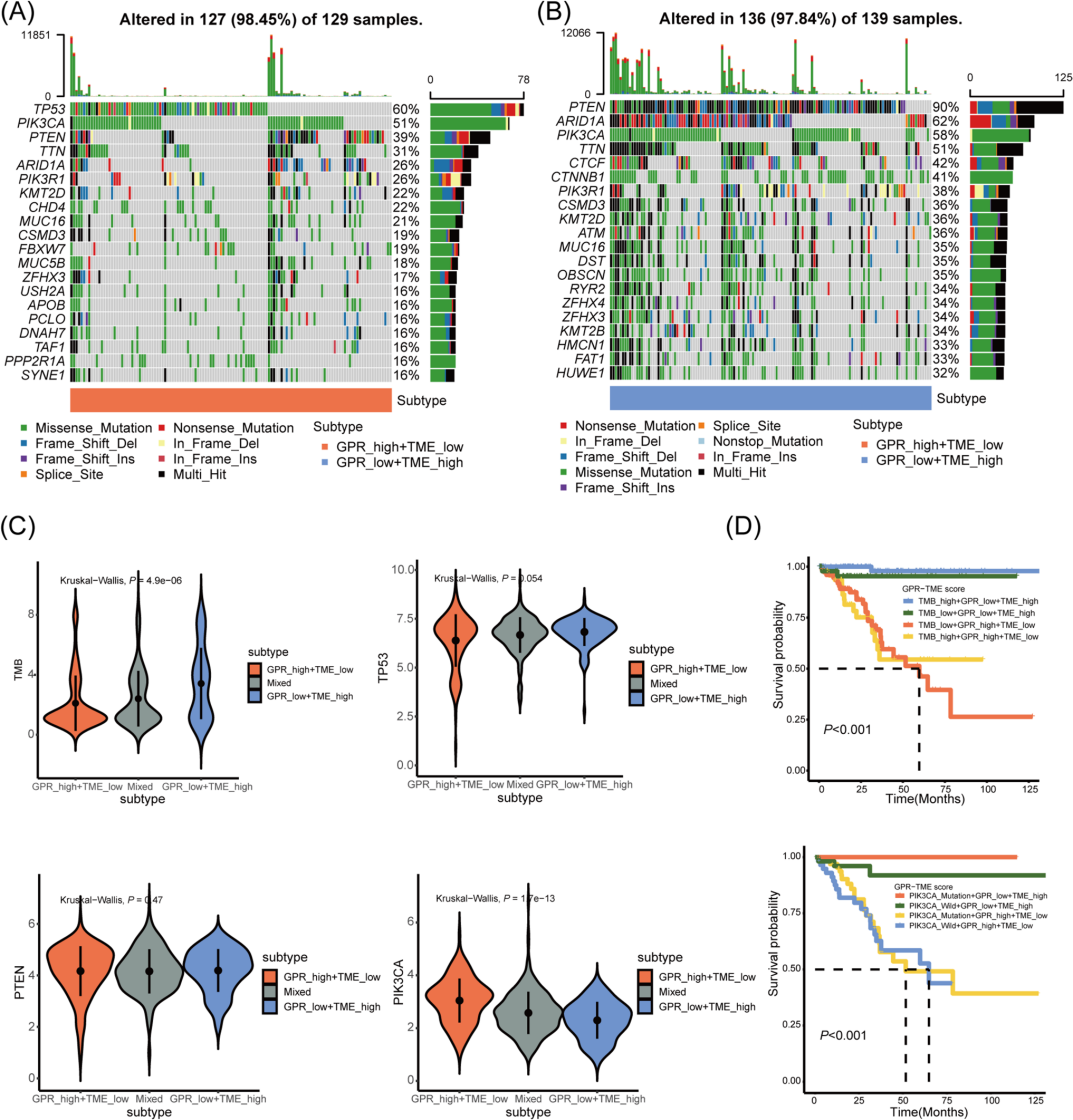
Association between tumor somatic mutations and GPR-TME classifier. The OncoPrint was constructed by the top 20 mutation genes between GPR_high + TME_low **A** and GPR_low + TME_high subgroups **B**. Each EC from an individual patient was represented in each column (TCGA-UCEC). **C** Comparison of TMB, TP53, PTEN, and PIK3CA expression among defined subgroups according to GPR-TME classifier. **D** K–M curves of EC patients divided by TMB (or PIK3CA status) and GPR-TME classifier. *GPR* G protein-coupled receptor, *TME* tumor environment, *TCGA-UCEC* the Cancer Genome Atlas-Uterine Corpus Endometrioid Carcinoma, *TMB* tumor mutational burden, *TP53* tumor protein 53, *PTEN* phosphatase and tensin homolog, *PIK3CA* phosphatidylinositol-4,5-bisphosphate 3-kinase catalytic subunit alpha

We next examined the tumor somatic alterations among different GPR-TME subgroups. The top 20 variant mutations in the TCGA-UCEC cohort were identified (Fig. 7A, B). The tumor somatic mutation landscapes differ between groups. TP53, PIK3CA, and PTEN mutations rank top three in the GPR_high + TME_low subgroup; while PTEN, ARID1A, and PIK3CA mutations rank top three in the GPR_low + TME_high subgroup. Compared to Fig. 7A, B, the GPR_low + TME_high subgroup was distinguished by a higher somatic mutation. Similarly, as depicted in Fig. 6C, the TMB in the GPR_low + TME_high subgroup was significantly higher than those in GPR_high + TME_low and Mixed subgroups. Further examination of the most prevalent mutations among subgroups revealed that PIK3CA expression varied among these subgroups (Fig. 7C). Detailed analysis was performed using K-M curves mixed with TMB (or PIK3CA status) and GPR-TME subgroups. However, neither TMB nor PIK3CA status could successfully optimize the predictive efficacy of the GPR-TME classifier (Fig. 7D).

### Distinct immune response profile in tumors among GPR-TME subgroups

**Fig. 8:**
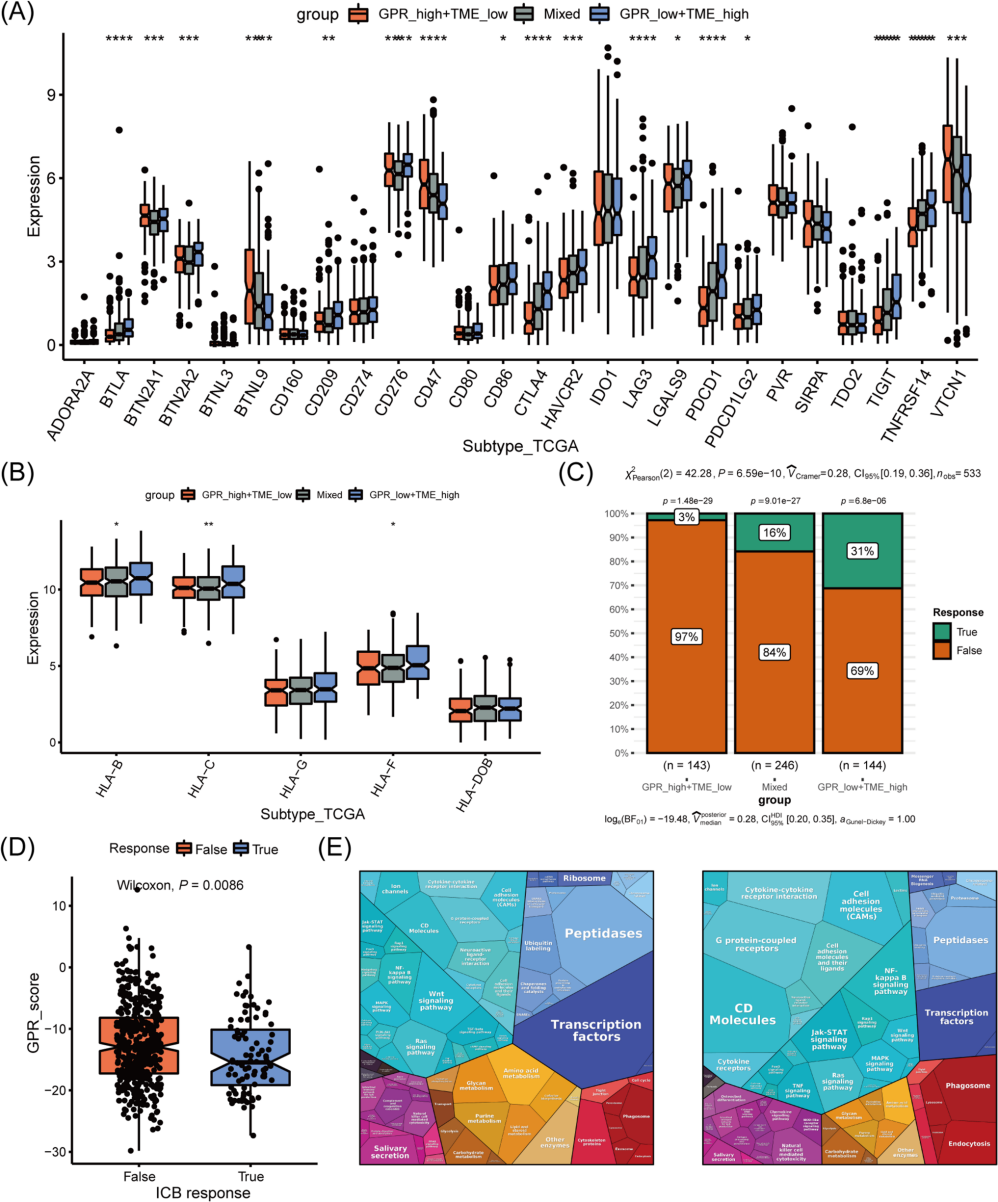
Comparison of immune-related markers in three subtypes and therapy response prediction based on GPR-TME classifier. **A** Comparison analysis of the inhibitory immune molecules among subgroups based on the GPR-TME classifier. **B** Comparison analysis of the HLA molecules among subgroups based on the GPR-TME classifier. **C** The different percentages of ICB responder among subgroups based on the GPR-TME classifier. **D** Comparison of GPR scores among patients with different ICB immunotherapy response status. **E** Functional analysis in GPR_high + TME_low (left) and responder (right) of patients under ICB immunotherapy illustrated using Proteomaps. *GPR* G protein-coupled receptor, *TME* tumor environment, *HLA* human leukocyte antigen, *ICB* immune checkpoint blockade

The immune response-associated genes among different subgroups were further investigated (Liu et al. 2021a). The GPR_low + TME_high subgroup had a higher expression of most of the inhibitory immune markers (including CTLA4, LAG3, and PDCD1; Fig. 8A) and human leukocyte antigen (HLA) markers (Fig. 8B) than the GPR_high + TME_low and Mixed subgroups. Given these findings, we tested whether the GPR-TME classifier could predict clinical responses in immunotherapies patients. TIDE for predicting immunotherapy response was used to solve this interrogatory. As depicted in Fig. 8C, the GPR_low + TME_high subgroup had the highest percentage (31%) of patients with therapeutic response to an immune checkpoint blockade (ICB), while the GPR_high + TME_low subgroup had only 3%. Patients with EC responding to ICB therapy showed statistically lower GPR scores (Fig. 8D). Similar patterns of Proteomaps are observed in the GPR_low + TME_high subgroup and ICB responder (Fig. 8E). These findings may suggest that the pretreatment GPR-TME classifier can depict the tumor immune microenvironment, thereby enhancing the EC patient's therapy responses prediction.

### Clinical validation for mRNA and protein expression of GRP-related gene

**Fig. 9:**
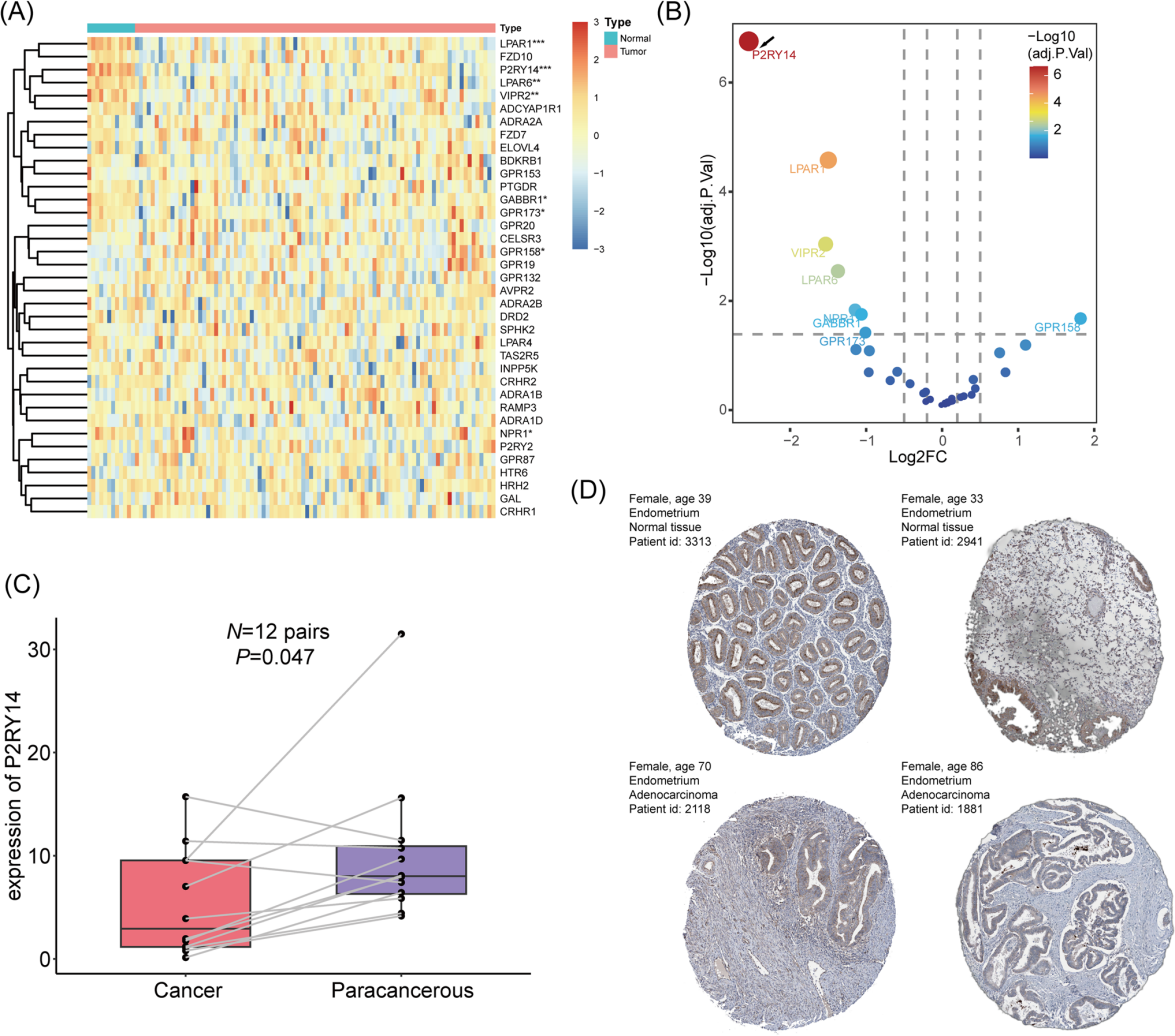
GEO filtration for DEGs of GPR-related genes and validation in clinical tissue samples. A Heatmap of DEGs of GPR-related genes from a GEO cohort (GSE17025). **B** Volcano plot of DEGs of GPR-related genes intersected by TCGA-UCEC and GEO (GSE17025) cohorts. **C** qRT-PCR of P2RY14 gene expression in EC samples (cancers vs. paracancerous, *n* = 12). **D** Protein expression of P2RY14 in EC and normal endometrium in the HPA database. Significance: *P < 0.05; ***P < 0.001. *GEO* Gene Expression Omnibus, *DEGs* differentially expressed genes, *GPR* G protein-coupled receptor, *TCGA-UCEC* the Cancer Genome Atlas-Uterine Corpus Endometrioid Carcinoma, *qRT-PCR* quantitative real time PCR, *EC* endometrioid carcinoma, *the HPA* the Human Protein Atlas

To further explore the potential GPR-related targets regulating the process of EC, the GEO dataset (GSE17025) was enrolled for crossed DEGs filtration. Interestingly, the purinergic receptor encoding gene P2RY14 presents significantly downregulated in EC tissues compared to normal endometrium tissues (Fig. 9A, B). qRT-PCRT’s results derived from twelve pairs of EC tissues and its paracancerous endometrium tissues also shows the similar results suggested by the crossed DEGs (Fig. 9C). In terms of protein level, HPA database was applied for inspecting the different protein expression of P2RY14. Conformably, the IHC staining from HPA database presents quite weak P2RY14 expression in EC glandular tissues compared to normal endometrium glandular (Fig. 9D).

## Discussion

The explosion of research on GPR and TME enhances our understanding of the possibility of combining these factors to predict cancer patients' prognosis and therapies (O’Hayre et al. 2014). However, few studies have integrated GPR and TME signatures to predict prognosis and therapeutic responses (Zhao et al. 2022). Signatures based on the combination of GPR and TME may enable clinical classification and therapeutic strategy optimization when targeting GPR combined with immunotherapy for EC treatment. Here, we systematically utilized the TCGA-UCEC cohort to assess the prognostic and therapeutic value of the GPR-TME classifier.

In this study, twenty GPR-related genes were enrolled in our risk signature, including adrenoceptor, prostaglandin receptors, vasoactive intestinal peptide (VIP) receptors, dopamine receptors, lysophosphatidic acid receptors, corticotropin-releasing hormone (CRH) receptors, purinergic receptors, bradykinin receptors, among others. Many of these novel targets have been studied in EC research. Dopamine receptor D2 (DRD2) overexpression in EC was significantly associated with grade, serous histology, stage, and worse progression-free survival and overall survival. While antagonizing DRD2 with ONC201 exhibited significant anti-tumorigenic effects in EC cells and an EC transgenic mouse model (Pierce et al. 2021). DRD2 was reported to promote malignant tumor progression by activating the oxygen-independent hypoxia-inducible factor-1α (HIF-1α) pathway in response to psychologic stress (Liu et al. 2021b). Similarly, CRHR1 was identified as an independent prognostic factor for EC in disease-free survival (DFS) and OS (Sato et al. 2014). A subfamily of adhesion GPRs in EC was recently thoroughly analyzed, and ADGRF1 was highlighted for its dual role in prognosis prediction and immune infiltrating evaluation (Lei et al. 2022). Purinergic receptors, the most common GPRs in the current model, play critical roles in various cancers. Purinergic receptor P2Y2 (P2RY2) activation has been reported to promote tumor cell proliferation via multiple downstream signaling pathways (Zaparte et al. 2021; Dong et al. 2022). The P2RY2 enhancer RNA (P2RY2e) has been validated as an estrogen-responsive eRNA and has been involved in the development of breast cancer and the progression of bladder cancer (Ding et al. 2018). Given that EC is an estrogen-related malignancy, we suggested that enhanced expression of P2RY2 induced by unopposed estrogenic stimulation may also promote the progression of EC. Conversely, down-regulation of P2RY14 in head and neck squamous cell carcinoma (HNSC) patients was associated with poor prognosis and reduced immune infiltration, indicating a conversion from immune-dominant to metabolic-dominant status (Li et al. 2021). The down-regulation of P2RY14 in TME of EC needs further investigation, as confirmed by the GEO dataset and clinical tissue samples (Fig. 9). Many GPRs in the galectin (GAL) subfamily were associated with immune suppression and regulated cytotoxicity T cell fate (Cagnoni et al. 2021; Yang et al. 2021), highlighting potential therapeutic targets for cancer immunotherapy. GPR132 mediates tumor-macrophage interactions to promote the alternatively activated M2-like phenotype by detecting lactate in the acidic tumor microenvironment. This facilitates cancer cell adhesion, migration, and invasion (Chen et al. 2017). Overall, research focusing on GPR functions and their interaction with tumor immune microenvironment has the potential to broaden the horizons of cancer immunology.

For the other part of the classifier, the TME score also exerts its critical role in cancer control. This model identified CD8 T cells, memory-activated CD4 T cells, activated NK cells, Tfh cells, and plasma cells as EC TME protectors. The anti-cancer effect of CD8 T cells, activated CD4 T cells, and activated NK cells have been verified in multiple cancers. Since in-depth research has been conducted on antitumor immunity, the function of Tfh cells and plasma cells in TME has emerged gradually. Tfh cells not only function in shaping B cell response during germinal center formation (Hollern et al. 2019) but also exert an antitumor immune effect in a CD8^+^-dependent manner. Tfh cells restore the exhausted T cells’ cytotoxic functions by producing interleukin-21 (IL-21), thereby enhancing the anti-PD-L1/PD-1 efficacy (Niogret et al. 2021). Plasma cells secrete specific antibodies into tumor niche as executors of humoral immunity. A recent study in EC has demonstrated that polymeric immunoglobulin receptor (pIgR) dependent, antigen-independent IgA occupancy triggers the activation of interferon (IFN) and tumor necrosis factor (TNF) signaling pathway in tumor cells. This activation is accompanied by apoptotic and endoplasmic reticulum stress downstream while hindering the DNA repair mechanisms (Mandal et al. 2022). After reannotating the EC scRNA sequencing dataset with MSI‑H/MMR‑d (Guo et al. 2022), the GPR score was significantly elevated in pDC and NKT cells. The intercellular communication analysis revealed impaired cell-chatting function in these GPR-elevated cells. Jiang et al. recently depicted that single-cell profiling of the immune atlas of tumor-infiltrating lymphocytes (TILs) in EC (Jiang et al. 2022). There was a cluster of NKT cells in their TILs atlas; however, the specific functions of this cluster have not been thoroughly analyzed yet.

Throughout the study, EC patients in the GPR_low + TME_high subgroup exhibited the most favorable prognosis and clinical responses to ICB treatment. The time-dependent ROC curves confirmed the sensibility and specificity of this risk signature. When merged for simplification, the GPR-TME classifier was identified as an independent prognostic factor for EC patients. This classifier’s OS predictive value was independent of the patient’s age, tumor grade, and stage, indicating a stable prediction efficiency. Moreover, multiple algorithms methods for functional annotation revealed different biological process enrichment among GPR-TME subgroups. This may imply that the host tumor profiles of the various GPR-TME subgroups share certain common characteristics. The results of tumor somatic mutations analysis revealed subtle correlations between the molecular subtyping of EC and the GPR-TME subgroup. The frequent TP53 mutations observed in the GPR_high + TME_low subgroup suggest a large body of copy number high (CN-H) molecular subtyping in this cluster. This corresponds to higher cell proliferation potentials and poor prognosis (Kandoth et al. 2013; Talhouk et al. 2015). Conversely, the GPR_low + TME_high subgroup indicated more common PTEN and ARID1A mutations suggesting potential association with other molecular subtypes (Kandoth et al. 2013; Stelloo et al. 2016). Further studies are needed to elucidate this vague relationship.

Immunotherapy has presented clinical efficacy in some patients with gynecological malignancies (Taha et al. 2020), primarily employed for advanced and recurrent cases that have failed conventional treatment. Among the three major gynecological malignancies, EC is the most curable. Currently, the evidence of ICIs application in advanced/recurrent EC mainly comes from the KEYNOTE series clinical trials of Pembrolizumab (KEYNOTE-016, KEYNOTE-158, KEYNOTE-028) (Ott et al. 2017; Le et al. 2015; O'Malley et al. 2022). The objective response rate (ORR) for Pembrolizumab in EC patients with MSI-H/dMMR ranged from 53.0% to 57.1% in the KEYNOTE trials. Similarly, the ORR for Nivolumab and Dostarlimab-gxly in EC patients with dMMR was over 40% in the EAY131 (Azad et al. 2020) and GARNET (Oaknin et al. 2022b) studies. These studies provide additional support using PD-1/PD-L1 mAbs in previously treated patients with MSI-H/dMMR advanced/recurrent EC. Notably, MSI-H/dMMR patients are the primary recipients of ICIs. However, only approximately 25% of EC patients have MSI-H/dMMR aberrance (Stelloo et al. 2016). This indicates that it is unclear whether immunotherapy will benefit the remaining patients. In our GPR-TME classifier, over 30% of patients in the GPR_low + TME_high subgroup were successfully predicted to benefit from immunotherapy regardless of the molecular subtyping. In contrast, merely 3% of GPR_high + TME_low patients were predicted to respond to ICB treatment, which is discouraged for immunotherapy.

Finally, we acknowledge certain limitations to this study. Firstly, the lack of survival data for EC necessitates the inclusion of more cohorts, particularly the in-house cohort, to further assess the performance of this classifier. Secondly, the GPR-TME signatures require additional validation using experimental methods.

## Conclusions

To our knowledge, this is the first comprehensive study conferring GPRs on a novel role in EC immune infiltration. In our study, depicting the integrated GPRs and immune cell landscape signatures within the TME may benefit the prediction of the EC patients’ prognosis and immunotherapy responses. It might be a potential method for prognosis assessment and stratification of EC patients for clinical management in practice.

### Supplementary Information

Below is the link to the electronic supplementary material.

**Supplementary Figure 1.** WGCNA for GPR-TME subgroups and PCA for GPR score evaluation. **A, B** The soft threshold for WGCNA (sft = 20). **C** Cluster dendrogram of five modules. **D** PCA evaluation for all GPR-related genes. **E** PCA evaluation for GPR score. WGCNA, weighted correlation network analysis; sft, soft threshold; GPR, G protein-coupled receptor; TME, tumor environment; PCA: principal component analysis. (TIF 1214 KB)

**Supplementary Figure 2.** Scrna Sequencing Analysis For One Case Of MSI‑H/MMR‑D EC Sample. (A, B) PCA For The MSI‑H/MMR‑D EC Sample. (C) UMAP Of 11 Clusters Of Cells For The MSI‑H/MMR‑D EC Sample. (D, E) Reannotation Of 11 Clusters Of Cells For The MSI‑H/MMR‑D EC Sample. Scrna, Single Cell RNA; MSI‑H/MMR‑D, Micro Satellite Instability-High/Mis-Match Repair-Deficiency; EC, Endometrial Carcinoma; PCA, Principal Component Analysis; UMAP, Uniform Manifold Approximation And Projection. (TIF 1326 KB)

Supplementary file3 (DOCX 17 KB)

## Data Availability

The RNA-seq data and corresponding clinical information were observed from TCGA (https://portal.gdc.cancer.gov/) and GEO (https://www.ncbi.nlm.nih.gov/geo/); scRNA sequencing data were obtained from GEO (https://www.ncbi.nlm.nih.gov/geo/). The accession number(s) can be found in the article/Supplementary Material.

## Abbreviations

EC: Endometrial carcinoma
GPRs: G protein-coupled receptors
LASSO: Least Absolute Shrinkage and Selection Operator
DEGs: Differentially expressed genes
TCGA: The Cancer Genome Atlas
WGCNA: Weighted correlation network analysis
TIDE: Tumor Immune Dysfunction and Exclusion
TMB: Tumor mutation burden
MSI: Microsatellite instability
HCC: Hepatocellular carcinoma
SPL: S1P lyase
LPI: Sphingolipids
LPG: Glycerophospholipids
A2AR: A2A receptors
DCs: Dendritic cells
PD-L1: Programmed cell death ligand 1
LUAD: Lung adenocarcinoma
ICIs: Immune checkpoint inhibitors
LPAR6: Lysophosphatidic acid receptor 6
scRNA: Single-cell RNA
MSI-H/MMR-d: Micro satellite instability-high/mis-match repair-deficiency
GEO: Gene Expression Omnibus
TIP: Tracking Tumor Immunophenotype
MSigDB: Molecular Signatures Database
TCGA-UCEC: The Cancer Genome Atlas-Uterine Corpus Endometrioid Carcinoma
TME-I: Tumor immune microenvironment
ssGSEA: Single-sample gene set enrichment analysis
NK: Natural killer
Tfh: Follicular helper T
OS: Overall survival
pDCs: Plasmacytoid DCs
PCA: Principal component analysis
ROC: Receiver operating characteristic
AUC: Area under the curve
CI: Confidence interval
HLA: Human leukocyte antigen
ICB: Immune checkpoint blockade
VIP: Vasoactive intestinal peptide
CRH: Corticotropin-releasing hormone
DRD2: Dopamine receptor D2
HIF-1α: Hypoxia-inducible factor-1α
DFS: Disease-free survival
P2RY2: Purinergic receptor P2Y2
HNSC: Head and neck squamous cell carcinoma
GAL: Galectin
IL-21: Interleukin-21
pIgR: Polymeric immunoglobulin receptor
IFN: Interferon
TNF: TILs: Tumor-infiltrating lymphocytes
CN-H: Copy number high
ORR: Objective response rate
CPTAC: Clinical Proteomic Tumor Analysis Consortium

## Declarations

### Human and animal participants

This article does not contain any studies with human participants or animals performed by any of the authors.

## References

[CR1] Azad NS, Gray RJ, Overman MJ, Schoenfeld JD, Mitchell EP, Zwiebel JA (2020). Nivolumab is effective in mismatch repair-deficient noncolorectal cancers: results from arm Z1D-A subprotocol of the NCI-MATCH (EAY131) Study. J Clin Oncol.

[CR2] Bai R, Zhang J, He F, Li Y, Dai P, Huang Z (2022). GPR87 promotes tumor cell invasion and mediates the immunogenomic landscape of lung adenocarcinoma. Commun Biol.

[CR3] Bar-Shavit R, Maoz M, Kancharla A, Nag JK, Agranovich D, Grisaru-Granovsky S (2016). G protein-coupled receptors in cancer. Int J Mol Sci.

[CR4] Bokhman JV (1983). Two pathogenetic types of endometrial carcinoma. Gynecol Oncol.

[CR5] Brinton LA, Felix AS, McMeekin DS, Creasman WT, Sherman ME, Mutch D (2013). Etiologic heterogeneity in endometrial cancer: evidence from a Gynecologic Oncology Group trial. Gynecol Oncol.

[CR6] Brown TP, Bhattacharjee P, Ramachandran S, Sivaprakasam S, Ristic B, Sikder MOF (2020). The lactate receptor GPR81 promotes breast cancer growth via a paracrine mechanism involving antigen-presenting cells in the tumor microenvironment. Oncogene.

[CR7] Cagnoni AJ, Giribaldi ML, Blidner AG, Cutine AM, Gatto SG, Morales RM (2021). Galectin-1 fosters an immunosuppressive microenvironment in colorectal cancer by reprogramming CD8(+) regulatory T cells. Proc Natl Acad Sci USA.

[CR8] Kandoth C, Schultz N, Cherniack AD, Akbani R, Liu Y, Cancer Genome Atlas Research N (2013). Integrated genomic characterization of endometrial carcinoma. Nature.

[CR9] Chen P, Zuo H, Xiong H, Kolar MJ, Chu Q, Saghatelian A (2017). Gpr132 sensing of lactate mediates tumor-macrophage interplay to promote breast cancer metastasis. Proc Natl Acad Sci USA.

[CR10] Chen B, Khodadoust MS, Liu CL, Newman AM, Alizadeh AA (2018). Profiling tumor infiltrating immune cells with CIBERSORT. Methods Mol Biol.

[CR11] Crosbie EJ, Kitson SJ, McAlpine JN, Mukhopadhyay A, Powell ME, Singh N (2022). Endometrial cancer. Lancet.

[CR12] Day RS, McDade KK, Chandran UR, Lisovich A, Conrads TP, Hood BL (2011). Identifier mapping performance for integrating transcriptomics and proteomics experimental results. BMC Bioinformatics.

[CR13] Ding M, Zhan H, Liao X, Li A, Zhong Y, Gao Q (2018). Enhancer RNA—P2RY2e induced by estrogen promotes malignant behaviors of bladder cancer. Int J Biol Sci.

[CR14] Dong CR, Hu DX, Liu SC, Luo HL, Zhang WJ (2022). AKT/GSK-3beta/VEGF signaling is involved in P2RY2 activation-induced the proliferation and metastasis of gastric cancer. Carcinogenesis.

[CR15] Dou Y, Kawaler EA, CuiZhou D, Gritsenko MA, Huang C, Blumenberg L (2020). Proteogenomic characterization of endometrial carcinoma. Cell.

[CR16] Ge YJ, Liao QW, Xu YC, Zhao Q, Wu BL, Ye RD (2020). Anti-inflammatory signaling through G protein-coupled receptors. Acta Pharmacol Sin.

[CR17] Guo YE, Liu Y, Zhang W, Luo H, Shu P, Chen G (2022). The clinicopathological characteristics, prognosis and immune microenvironment mapping in MSI-H/MMR-D endometrial carcinomas. Discov Oncol.

[CR18] He YY, Cai B, Yang YX, Liu XL, Wan XP (2009). Estrogenic G protein-coupled receptor 30 signaling is involved in regulation of endometrial carcinoma by promoting proliferation, invasion potential, and interleukin-6 secretion via the MEK/ERK mitogen-activated protein kinase pathway. Cancer Sci.

[CR19] He YY, Du GQ, Cai B, Yan Q, Zhou L, Chen XY (2012). Estrogenic transmembrane receptor of GPR30 mediates invasion and carcinogenesis by endometrial cancer cell line RL95-2. J Cancer Res Clin Oncol.

[CR20] He J, Gao R, Meng M, Yu M, Liu C, Li J (2021). Lysophosphatidic Acid Receptor 6 (LPAR6) Is a potential biomarker associated with lung adenocarcinoma. Int J Environ Res Public Health.

[CR21] He J, Meng M, Wang H (2022). A novel prognostic biomarker LPAR6 in hepatocellular carcinoma via associating with immune infiltrates. J Clin Transl Hepatol.

[CR22] Hollern DP, Xu N, Thennavan A, Glodowski C, Garcia-Recio S, Mott KR (2019). B Cells and T follicular helper cells mediate response to checkpoint inhibitors in high mutation burden mouse models of breast cancer. Cell.

[CR23] Huvila J, Pors J, Thompson EF, Gilks CB (2021). Endometrial carcinoma: molecular subtypes, precursors and the role of pathology in early diagnosis. J Pathol.

[CR24] Jiang P, Gu S, Pan D, Fu J, Sahu A, Hu X (2018). Signatures of T cell dysfunction and exclusion predict cancer immunotherapy response. Nat Med.

[CR25] Jiang F, Jiao Y, Yang K, Mao M, Yu M, Cao D (2022). Single-cell profiling of the immune atlas of tumor-infiltrating lymphocytes in endometrial carcinoma. Cancers (basel).

[CR26] Kuo CC, Wu JY, Wu KK (2022). Cancer-derived extracellular succinate: a driver of cancer metastasis. J Biomed Sci.

[CR27] Langfelder P, Horvath S (2008). WGCNA: an R package for weighted correlation network analysis. BMC Bioinform.

[CR28] Le DT, Uram JN, Wang H, Bartlett BR, Kemberling H, Eyring AD (2015). PD-1 blockade in tumors with mismatch-repair deficiency. N Engl J Med.

[CR29] Lei P, Wang H, Yu L, Xu C, Sun H, Lyu Y (2022). A correlation study of adhesion G protein-coupled receptors as potential therapeutic targets in Uterine Corpus Endometrial cancer. Int Immunopharmacol.

[CR30] Li Q, Xu L, Li Y, Yang R, Qiao Q, Wang Y (2021). P2RY14 is a potential biomarker of tumor microenvironment immunomodulation and favorable prognosis in patients with head and neck cancer. Front Genet.

[CR31] Li H, Wang J, Li L, Zhao L, Wang Z (2023). Expression of EMT-related genes in lymph node metastasis in endometrial cancer: a TCGA-based study. World J Surg Oncol.

[CR32] Liberzon A, Birger C, Thorvaldsdottir H, Ghandi M, Mesirov JP, Tamayo P (2015). The Molecular Signatures Database (MSigDB) hallmark gene set collection. Cell Syst.

[CR33] Liebermeister W, Noor E, Flamholz A, Davidi D, Bernhardt J, Milo R (2014). Visual account of protein investment in cellular functions. Proc Natl Acad Sci USA.

[CR34] Liu JH, Meng HY, Nie SP, Sun Y, Jiang PP, Li SY (2020). Identification of a prognostic signature of epithelial ovarian cancer based on tumor immune microenvironment exploration. Genomics.

[CR35] Liu JH, Chen C, Wang YC, Qian C, Wei JT, Xing Y (2021). Comprehensive of N1-methyladenosine modifications patterns and immunological characteristics in ovarian cancer. Front Immunol.

[CR36] Liu H, Yang J, Zhang Y, Han J, Yang Y, Zhao Z (2021). Psychologic stress drives progression of malignant tumors via DRD2/HIF1alpha signaling. Cancer Res.

[CR37] Liu JH, Geng R, Ni SM, Cai LX, Yang S, Shao F (2022). Pyroptosis-related lncRNAs are potential biomarkers for predicting prognoses and immune responses in patients with UCEC. Mol Ther Nucleic Acids.

[CR38] Makker V, Rasco D, Vogelzang NJ, Brose MS, Cohn AL, Mier J (2019). Lenvatinib plus pembrolizumab in patients with advanced endometrial cancer: an interim analysis of a multicentre, open-label, single-arm, phase 2 trial. Lancet Oncol.

[CR39] Mandal G, Biswas S, Anadon CM, Yu X, Gatenbee CD, Prabhakaran S (2022). IgA-dominated humoral immune responses govern patients' outcome in endometrial cancer. Cancer Res.

[CR40] McAlpine J, Leon-Castillo A, Bosse T (2018). The rise of a novel classification system for endometrial carcinoma; integration of molecular subclasses. J Pathol.

[CR41] Mori D, Tsujikawa T, Sugiyama Y, Kotani SI, Fuse S, Ohmura G (2021). Extracellular acidity in tumor tissue upregulates programmed cell death protein 1 expression on tumor cells via proton-sensing G protein-coupled receptors. Int J Cancer.

[CR42] Niogret J, Berger H, Rebe C, Mary R, Ballot E, Truntzer C (2021). Follicular helper-T cells restore CD8(+)-dependent antitumor immunity and anti-PD-L1/PD-1 efficacy. J Immunother Cancer.

[CR43] Oaknin A, Bosse TJ, Creutzberg CL, Giornelli G, Harter P, Joly F (2022). Endometrial cancer: ESMO Clinical Practice Guideline for diagnosis, treatment and follow-up. Ann Oncol.

[CR44] Oaknin A, Gilbert L, Tinker AV, Brown J, Mathews C, Press J (2022). Safety and antitumor activity of dostarlimab in patients with advanced or recurrent DNA mismatch repair deficient/microsatellite instability-high (dMMR/MSI-H) or proficient/stable (MMRp/MSS) endometrial cancer: interim results from GARNET-a phase I, single-arm study. J Immunother Cancer.

[CR45] O'Hayre M, Degese MS, Gutkind JS (2014). Novel insights into G protein and G protein-coupled receptor signaling in cancer. Curr Opin Cell Biol.

[CR46] O'Malley DM, Bariani GM, Cassier PA, Marabelle A, Hansen AR, De Jesus AA (2022). Pembrolizumab in patients with microsatellite instability-high advanced endometrial cancer: results from the KEYNOTE-158 Study. J Clin Oncol.

[CR47] Orduna-Castillo LB, Del-Rio-Robles JE, Garcia-Jimenez I, Zavala-Barrera C, Beltran-Navarro YM, Hidalgo-Moyle JJ (2022). Calcium sensing receptor stimulates breast cancer cell migration via the Gbetagamma-AKT-mTORC2 signaling pathway. J Cell Commun Signal.

[CR48] Ott PA, Bang YJ, Berton-Rigaud D, Elez E, Pishvaian MJ, Rugo HS (2017). Safety and antitumor activity of pembrolizumab in advanced programmed death ligand 1–positive endometrial cancer: results from the KEYNOTE-028 study. J Clin Oncol.

[CR49] Pierce SR, Fang Z, Yin Y, West L, Asher M, Hao T (2021). Targeting dopamine receptor D2 as a novel therapeutic strategy in endometrial cancer. J Exp Clin Cancer Res.

[CR50] Pillai S, Mahmud I, Mahar R, Griffith C, Langsen M, Nguyen J (2022). Lipogenesis mediated by OGR1 regulates metabolic adaptation to acid stress in cancer cells via autophagy. Cell Rep.

[CR51] Sato N, Takagi K, Suzuki T, Miki Y, Tanaka S, Nagase S (2014). Immunolocalization of corticotropin-releasing hormone (CRH) and its receptors (CRHR1 and CRHR2) in human endometrial carcinoma: CRHR1 as a potent prognostic factor. Int J Gynecol Cancer.

[CR52] Stelloo E, Nout RA, Osse EM, Jurgenliemk-Schulz IJ, Jobsen JJ, Lutgens LC (2016). Improved risk assessment by integrating molecular and clinicopathological factors in early-stage endometrial cancer-combined analysis of the PORTEC cohorts. Clin Cancer Res.

[CR53] Sun C, Wang B, Hao S (2022). Adenosine-A2A receptor pathway in cancer immunotherapy. Front Immunol.

[CR54] Sung H, Ferlay J, Siegel RL, Laversanne M, Soerjomataram I, Jemal A (2021). Global Cancer Statistics 2020: GLOBOCAN Estimates of Incidence and Mortality Worldwide for 36 Cancers in 185 Countries. CA Cancer J Clin.

[CR55] Taha T, Reiss A, Amit A, Perets R (2020). Checkpoint inhibitors in gynecological malignancies: are we there yet?. BioDrugs.

[CR56] Talhouk A, McConechy MK, Leung S, Li-Chang HH, Kwon JS, Melnyk N (2015). A clinically applicable molecular-based classification for endometrial cancers. Br J Cancer.

[CR57] Talhouk A, McConechy MK, Leung S, Yang W, Lum A, Senz J (2017). Confirmation of ProMisE: a simple, genomics-based clinical classifier for endometrial cancer. Cancer.

[CR58] Uranbileg B, Kurano M, Kano K, Sakai E, Arita J, Hasegawa K (2022). Sphingosine 1-phosphate lyase facilitates cancer progression through converting sphingolipids to glycerophospholipids. Clin Transl Med.

[CR59] Wang H, Wang X, Xu L, Zhang J, Cao H (2020). High expression levels of pyrimidine metabolic rate-limiting enzymes are adverse prognostic factors in lung adenocarcinoma: a study based on The Cancer Genome Atlas and Gene Expression Omnibus datasets. Purinergic Signal.

[CR60] Xu L, Deng C, Pang B, Zhang X, Liu W, Liao G (2018). TIP: a web server for resolving tumor immunophenotype profiling. Cancer Res.

[CR61] Yang R, Sun L, Li CF, Wang YH, Yao J, Li H (2021). Galectin-9 interacts with PD-1 and TIM-3 to regulate T cell death and is a target for cancer immunotherapy. Nat Commun.

[CR62] Zaparte A, Cappellari AR, Brandao CA, de Souza JB, Borges TJ, Kist LW (2021). P2Y(2) receptor activation promotes esophageal cancer cells proliferation via ERK1/2 pathway. Eur J Pharmacol.

[CR63] Zhang F, Peng L, Huang Y, Lin X, Zhou L, Chen J (2019). Chronic BDE-47 exposure aggravates malignant phenotypes and chemoresistance by activating ERK through ERalpha and GPR30 in endometrial carcinoma. Front Oncol.

[CR64] Zhang XW, Li L, Hu WQ, Hu MN, Tao Y, Hu H (2022). Neurokinin-1 receptor promotes non-small cell lung cancer progression through transactivation of EGFR. Cell Death Dis.

[CR65] Zhao L, Zhang H, Liu X, Xue S, Chen D, Zou J (2022). TGR5 deficiency activates antitumor immunity in non-small cell lung cancer via restraining M2 macrophage polarization. Acta Pharm Sin B.

[CR66] Zhou Y, Zhou B, Pache L, Chang M, Khodabakhshi AH, Tanaseichuk O (2019). Metascape provides a biologist-oriented resource for the analysis of systems-level datasets. Nat Commun.

