# Supplementary figures and images for: The combined signatures of G protein-coupled receptor family and immune landscape provide a prognostic and therapeutic biomarker in endometrial carcinoma
Source: J Cancer Res Clin Oncol. 2023 Aug 16;149(16):14701–19. doi: 10.1007/s00432-023-05270-4 (PMC10602984; doi:10.1007/s00432-023-05270-4)

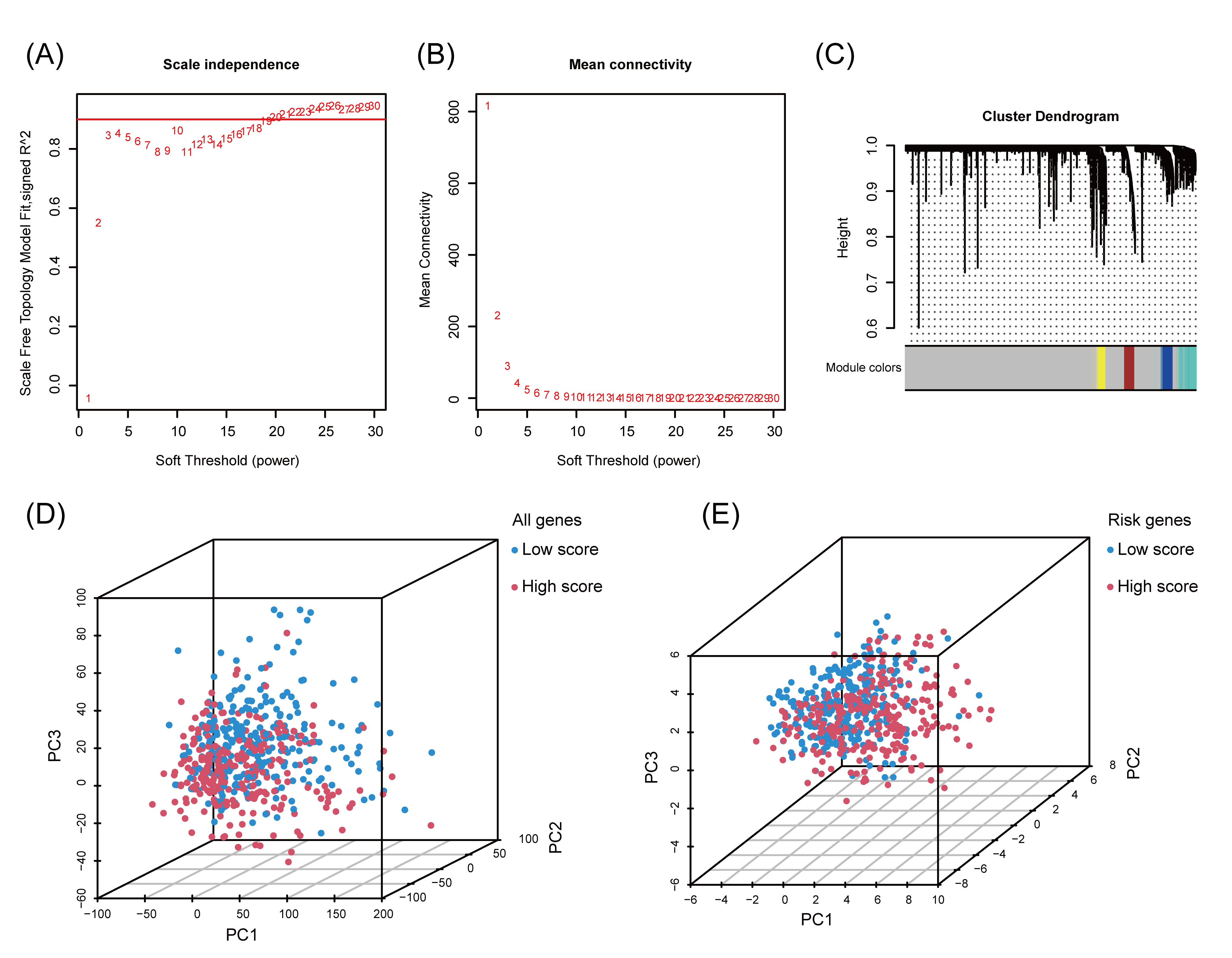

Supplement: Supplementary file 1 — Supplementary Figure 1. WGCNA for GPR-TME subgroups and PCA for GPR score evaluation. A, B The soft threshold for WGCNA (sft = 20). C Cluster dendrogram of five modules. D PCA evaluation for all GPR-related genes. E PCA evaluation for GPR score. WGCNA, weighted correlation network analysis; sft, soft threshold; GPR, G protein-coupled receptor; TME, tumor environment; PCA: principal component analysis. (TIF 1214 KB) [file 432_2023_5270_MOESM1_ESM.tif]

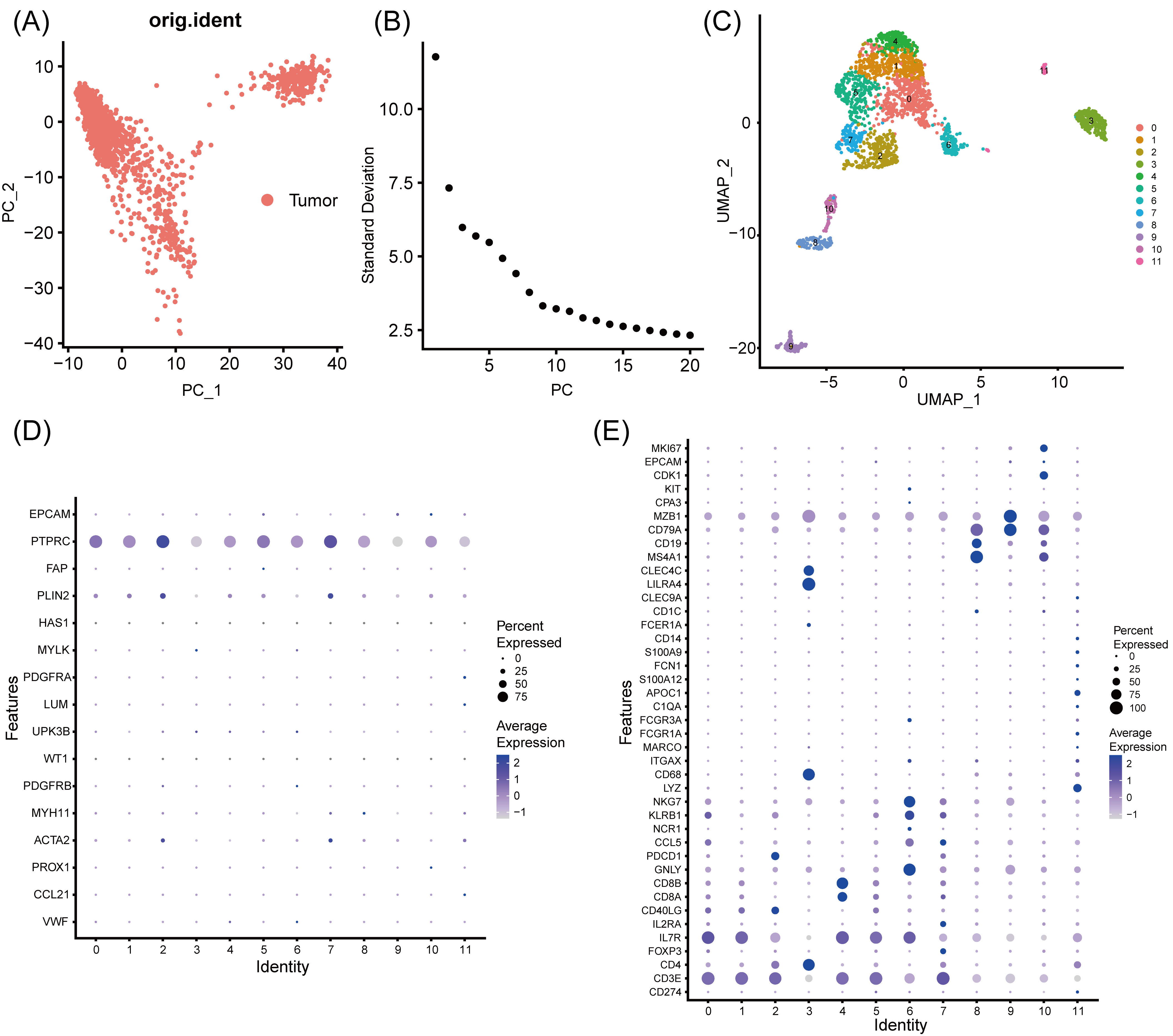

Supplement: Supplementary file 2 — Supplementary Figure 2. Scrna Sequencing Analysis For One Case Of MSI‑H/MMR‑D EC Sample. (A, B) PCA For The MSI‑H/MMR‑D EC Sample. (C) UMAP Of 11 Clusters Of Cells For The MSI‑H/MMR‑D EC Sample. (D, E) Reannotation Of 11 Clusters Of Cells For The MSI‑H/MMR‑D EC Sample. Scrna, Single Cell RNA; MSI‑H/MMR‑D, Micro Satellite Instability-High/Mis-Match Repair-Deficiency; EC, Endometrial Carcinoma; PCA, Principal Component Analysis; UMAP, Uniform Manifold Approximation And Projection. (TIF 1326 KB) [file 432_2023_5270_MOESM2_ESM.tif]
