# Supplementary material for: The combined signatures of G protein-coupled receptor family and immune landscape provide a prognostic and therapeutic biomarker in endometrial carcinoma
Source: J Cancer Res Clin Oncol. 2023 Aug 16;149(16):14701–19. doi: 10.1007/s00432-023-05270-4 (PMC10602984; doi:10.1007/s00432-023-05270-4)
Supplement: Supplementary file 3 — Supplementary file3 (DOCX 17 KB) [file 432_2023_5270_MOESM3_ESM.docx]

**Supplementary Table 1 Clinical characteristics of the UCEC patients in TCGA and CPTAC dataset**

|  |  | **TCGA dataset** | **CPTAC dataset** | ***P*** |
| --- | --- | --- | --- | --- |
| **No.** |  | 548 | 83 |  |
| **Age** | < 65 y | 312 (56.9%) | 55 (66.3%) | 0.108 |
|  | ≥65 y | 236 (43.1%) | 28 (33.7%) |  |
| **Race** | White | 374 (68.2%) | 52 (62.7%) | 0.005 |
|  | Black or African American | 109 (19.9%) | 2 (2.4%) |  |
|  | Asian | 20 (3.6%) | 1 (1.2%) |  |
|  | Others | 45 (8.2%) | 28 (33.7%) |  |
| **Grade** | G1+G2 | 221 (40.3%) | 75 (52.1%) | 0.005 |
|  | G3 | 327 (59.7%) | 8 (5.6%) |  |
| **Stage** | Stage Ⅰ+Ⅱ | 394 (71.9%) | 71 (85.5%) | 0.009 |
|  | Stage Ⅲ+Ⅳ | 154 (28.1%) | 12 (14.5%) |  |
| **Histologic_type** | Endometrioid | 385 (70.3%) | 75 (90.4%) | 0.007 |
|  | Non-endometrioid | 113 (20.6%) | 8 (9.6%) |  |
|  | Unknown | 50 (9.1%) | 0 (0.0%) |  |
| **Lymph_node_metastasis** | Positive | 75 (13.7%) | 7 (8.4%) | 0.758 |
|  | Negative | 423 (77.2%) | 45 (54.2%) |  |
|  | Unknown | 50 (9.1%) | 31 (37.3%) |  |
| **Myometrial_invasion** | <1/2 | 311 (56.8%) | 54 (65.1%) | 0.649 |
|  | ≥1/2 | 187 (34.1%) | 29 (34.9%) |  |
|  | Unknown | 50 (9.1%) | 0 (0.0%) |  |

TCGA: The Cancer Genome Atlas; CPTAC: Clinical Proteomic Tumor Analysis Consortium
